# High Potential for Anaerobic Microbial Sulfur Oxidation in Oil Sands Tailings Ponds

**DOI:** 10.3390/microorganisms9122529

**Published:** 2021-12-07

**Authors:** Sebastian Stasik, Juliane Schmidt, Katrin Wendt-Potthoff

**Affiliations:** Department of Lake Research, Helmholtz Centre for Environmental Research—UFZ, 04318 Leipzig, Germany; Sebastian.Stasik@uniklinikum-dresden.de (S.S.); juliane.schmidt19@gmx.de (J.S.)

**Keywords:** oil sands tailings ponds, anaerobic sulfur oxidation, sulfur-oxidizing bacteria, thiosulfate oxidation, microbial activity

## Abstract

The biogenic production of toxic H_2_S gas in sulfate-rich oil sands tailings ponds is associated with strong environmental concerns. Beside precipitation into sulfide minerals and chemical re-oxidation, microbial sulfur oxidation may catalyze sulfide re-cycling but potentially contributes to acid rock drainage (ARD) generation. To evaluate the microbial potential for sulfur oxidation, we conducted a microcosm-based pilot study with tailings of an active pond. Incubations were performed under oxic and anoxic conditions, with and without KNO_3_ as an electron acceptor and thiosulfate as a common substrate for microbial sulfur oxidation. The highest potentials of sulfur oxidation occurred in oxic assays (1.21 mmol L^−1^ day^−1^). Under anoxic conditions, rates were significantly lower and dominated by chemical transformation (0.09 mmol L^−1^ day^−1^; *p* < 0.0001). The addition of KNO_3_ to anoxic incubations increased microbial thiosulfate oxidation 2.5-fold (0.23 mmol L^−1^ day^−1^; *p* = 0.0474), with complete transformation to SO_4_^2−^ coupled to NO_3_^−^ consumption, pointing to the activity of sulfur-oxidizing bacteria (SOB) under nitrate-reducing conditions. Importantly, in the presence of KNO_3_, a decrease in sedimentary sulfides was associated with an increase in S^0^, which indicates the potential for microbially mediated oxidation of sulfide minerals and ARD generation. Furthermore, the comparative analysis of sediments from other anthropogenic aquatic habitats demonstrated high similarities with respect to viable SOB counts and corresponding activity rates.

## 1. Introduction

Oil sands tailings ponds are the primary settling basins for the storage and long-term containment of tailings produced during the extraction of bitumen from surface mining in northern Alberta (Canada) [1]. Due to the large scale of operations, oil sands development is associated with adverse environmental issues, such as the accumulation of toxic hydrocarbons (i.e., naphthenic acids) and the emission of biogenic greenhouse gases (primarily methane) [2,3].

In addition to methanogenesis, a diversity of indigenous microbial communities contribute to hydrocarbon degradation and drive elemental cycles of iron, nitrogen and sulfur [4]. Consequently, the microbial production of toxic H_2_S gas in sulfate-rich tailings poses a strong environmental and operational concern [1,4]. However, laboratory and field observations indicate that outgassing of H_2_S is effectively prevented by the immobilization of sulfide into the mineral phase of the ponds (i.e., in the form of FeS/FeS_2_) and/or by the biochemical re-oxidation to SO_4_^2−^ [3,4,5,6]. In line with this, viable cell counts and molecular analyses in anoxic tailings and oxic surface waters demonstrated high numbers of viable bacteria capable of utilizing reduced sulfur species (SOB) [7,8] and revealed the presence of bacterial taxa (i.e., *Thiobacillus*, *Epsilonproteobacteria* and *Halothiobacillus*) typically associated with the aerobic and anaerobic oxidation of reduced sulfur compounds. These findings have potential relevance for the generation of acid rock drainage (ARD) during pond reclamation [4,9,10]. However, while H_2_S/HS^−^ was shown to undergo a fast chemical oxidation upon contact with oxidized water layers or via the reaction with buried iron-(hydro)oxide in anoxic pond sediments [4,11], the potential activity of anaerobic microbial sulfide oxidation to sulfur cycling is less well characterized.

Depending on the prevalent oxidant-to-sulfide ratio, a variety of inorganic intermediate sulfur species (i.e., S^0^ and S_2_O_3_^2−^) may be produced during chemical or biological oxidation of sulfide in freshwater and marine sediments [12,13]. As a major oxidation product, thiosulfate (S_2_O_3_^2−^) can be used by many SOB to produce sulfate via the SOX pathway [14] under oxic conditions or in association with dissimilatory nitrate reduction (DNR) [15]. Organisms capable of DNR have been isolated from several human-made habitats such as oil fields, underground storage tanks and wastewater treatment plants, and the process has been applied technically in the coastal farming industry, for groundwater treatment or for sulfide and odor control of oil fields and sediments [16]. During landscape reclamation and associated formation of end pit lakes (EPL), nitrogen cycling will gradually become more important [17], and nitrate continuously generated by nitrification may serve as an electron acceptor for anaerobic microbial oxidation of reduced sulfur species. This process will control nitrogen and sulfide levels in the lakes but may ultimately lead to ARD generation.

In order to evaluate the microbial potential for the oxidation of reduced sulfur compounds in tailings material of an active pond, we performed a comprehensive microcosm-based assessment of sulfur oxidation rates under oxic and anoxic conditions. Thiosulfate, a common substrate oxidized by almost all sulfur lithotrophs, was chosen as a model compound in laboratory incubations. In addition, KNO_3_ was used as an electron acceptor for anaerobic sulfur oxidation in a subset of microcosms. To put the findings from tailings material into context, we set up comparable incubations with sediment from two other human-made and sulfur-rich water bodies, namely, a eutrophic pre-dam in a mountainous catchment and an acid pit lake resulting from opencast lignite mining. All of these habitats are at least temporarily stratified and provide an oxic–anoxic boundary. We demonstrate a strong indigenous potential for aerobic and anaerobic microbial sulfur oxidation, coupled to nitrate respiration, which is comparable to other sulfur-rich aquatic habitats. In addition, the assessment of total reduced inorganic sulfur (TRIS) in anoxic assays with the addition of nitrate points to the microbially catalyzed oxidation of sedimentary sulfides, which might become especially important in the light of potential ARD generation in future end pit lakes.

## 2. Materials and Methods

### 2.1. Samples

The tailings sample was collected in October 2010 from an active tailings pond (West In-Pit; Syncrude Canada Ltd., Fort McMurray, AB, Canada) using a piston sampler, as described previously [6]. For shipment, the samples were filled into 500 mL plastic Nalgene bottles. All bottles were filled to the top without any headspace. Samples were sealed and shipped within 2 weeks, darkened and cooled to minimize biogeochemical alterations. Study sites for comparative analysis comprised the acidic (pH 2.6) pit lake Mining Lake 111 (ML 111; former lignite mine, Lusatia, Germany) and the stratified eutrophic Hassel pre-dam (Rappbode reservoir system, Harz Mountains, Germany). Anoxic sediment samples from both lakes were collected using a UWITEC gravity corer. They were filled into sterile plastic bags without air bubbles, and these bags were placed into Anaerocult^®^ bags with gas generators (Merck, Darmstadt, Germany) to stabilize anoxia.

### 2.2. Most Probable Numbers (MPN)

MPN to enumerate viable cells of SOB were performed as serial dilutions in deep-well plates with eight parallels, as described previously [18]. Selective media for SOB were used following [19] with original salt concentrations for the Hassel pre-dam and ML 111 [g L^−1^]: NaCl (0.1), MgCl·6H_2_O (0.2), NH_4_Cl (0.1), KH_2_PO_4_ (0.1), KCl (0.1), CaCl_2_·2H_2_O (0.2). Elevated concentrations of [g L^−1^] NaCl (1.0), MgCl·6H_2_O (3.0), NH_4_Cl (0.3), KH_2_PO_4_ (0.2), KCl (0.3) and CaCl_2_·2H_2_O (0.15) were used for West In-Pit because of its high salt concentrations in situ [6]. Thiosulfate concentration was 10 mmol L^−1^, and 1 mL of trace element solution SL12 and 1 mL of vitamin solution were added per liter of medium. Media for the Hassel pre-dam and West In-Pit were adjusted to pH 7, and bromocresol purple (5 mg L^−1^) was added as a pH indicator. Media for ML 111 were adjusted to pH 4 to reflect the acidic condition of the lake, and bromophenol blue (5 mg L^−1^) was used to indicate further acidification as a result of SOB growth. Final media were sterilized by filtration (0.2 µm). Plates were incubated in the dark at 26 °C for six weeks. MPN and their confidence intervals were calculated using the program developed by Klee [20].

### 2.3. Thiosulfate Oxidation Potentials

Thiosulfate oxidation potentials were quantified in batch slurries containing a 1:1 (*v*/*v*) ratio of sediment sample to added liquids. Liquids consisted of the mineral media used for MPN quantification but without pH indicators, a trace element solution, SL12 [19], and Na_2_S_2_O_3_·5H_2_O (thiosulfate), with a final concentration of 3.75 mmol L^−1^. Assays (70 mL) were incubated under aerobic and anaerobic conditions in 100 mL Erlenmeyer flasks or 125 mL serum bottles with butyl rubber stoppers on a shaker (100 rpm) for two weeks. Anoxic media in serum bottles were extensively bubbled with dinitrogen gas before autoclaving, and all further handling took place in an anaerobic chamber (Bactron, Sheldon, OR, USA, gas phase 95% N_2_ and 5% H_2_). Autoclaved assays incubated in the same way served as abiotic controls. All assays were conducted in triplicate in the dark at 20 °C. KNO_3_ at a final concentration of 4 mmol L^−1^ was added to half of the anaerobic assays to account for nitrate-dependent thiosulfate oxidation. To account for a potential effect of low pH for rates of thiosulfate oxidation in ML 111, incubations with sediments of the acidic pit lake were performed at pH 4 and pH 6. Concentrations of thiosulfate, sulfate and nitrate were measured daily by ion chromatography (Dionex ICS-3000). For this procedure, 4 mL of slurry was sampled aseptically with a syringe. Pore water was obtained by centrifugation (10 min; 13,750 rpm) and subsequent filtration (0.2 µm; PTFE). Rates were calculated as linear regressions of thiosulfate decrease over time.

### 2.4. Total Reduced Inorganic Sulfur (TRIS)

TRIS was analyzed in material prior to and at the end of incubation, according to a sequential extraction method following Canfield [21] and Fossing and Jørgensen [22]. The extracted fractions of acid-volatile sulfide (AVS; H_2_S/HS^−^ and FeS), chromium-reducible sulfide (CRS; FeS_2_) and dimethylformamide-extractable sulfur (DMFS; mainly S^0^) were measured polarographically, as described elsewhere [23].

### 2.5. Statistical Analysis

Variables between groups were compared using a two-sided Student t-test or the nonparametric Mann–Whitney U test. Calculations were conducted using Prism 5 (GraphPad, La Jolla, CA, USA). Values of *p* < 0.05 were considered significant.

## 3. Results

### 3.1. Thiosulfate Oxidation Potentials in Tailings Material

**Figure 1:**
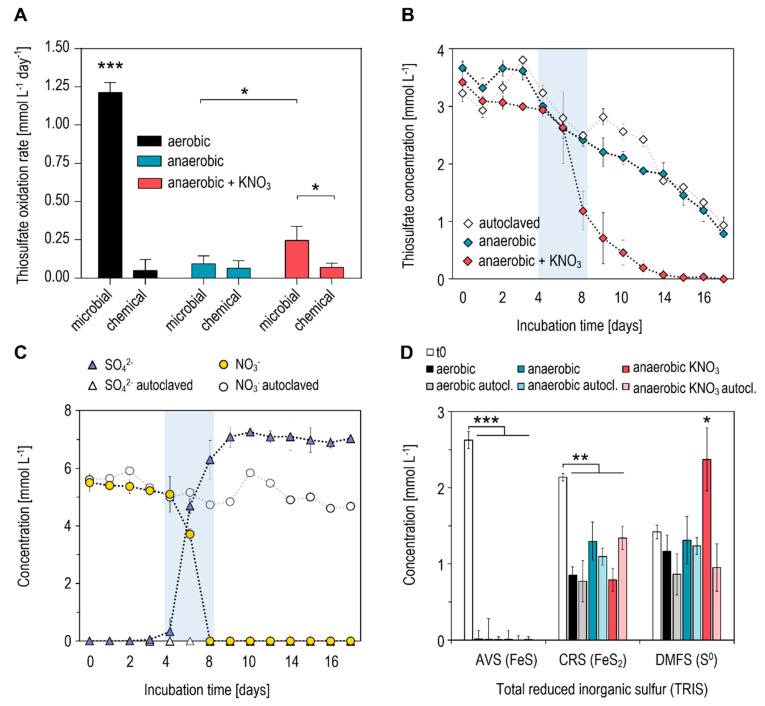
Microcosm-based assessment of thiosulfate oxidation potentials in tailings material of an active oil sands tailings pond. (**A**) Thiosulfate oxidation rates measured in aerobic and anaerobic assays. “Chemical” rates refer to oxidation potentials measured in autoclaved microcosms. (**B**) Transformation of thiosulfate under anaerobic conditions. (**C**) Concentrations of sulfate and nitrate in anaerobic microcosms with KNO_3_. (**D**) Concentrations of total reduced inorganic sulfur (TRIS) fractions prior to (t0) and at the end (14 days) of tailings incubation under various conditions. Analyzed TRIS fractions are: AVS (acid-volatile sulfide; FeS), CRS (chromium-reducible sulfide; FeS_2_) and DMFS (dimethylformamide-extractable sulfur; S^0^). All data points and bars represent mean values of triplicate incubations ± standard deviation. * *p* < 0.05; ** *p* < 0.01; *** *p* < 0.001.

The highest potential for microbial thiosulfate oxidation in tailings material was measured in aerobic incubations with a rate of 1.21 ± 0.09 mmol L^−1^ day^−1^ (Figure 1A). Under these conditions, chemical transformation (quantified in autoclaved assays) accounted for ~4% (0.05 mmol L^−1^ day^−1^) of thiosulfate turnover. In anaerobic microcosms, the rate of thiosulfate transformation was significantly lower (0.09 ± 0.08 mmol L^−1^ day^−1^; *p* < 0.0001) and dominated by chemical reactions (~78%), as seen in the autoclaved controls (Figure 1A,B). Maximum SO_4_^2−^ accumulation in these assays amounted to 2.81 mmol L^−1^ (not shown). Hence, considering a theoretical production of 7.5 mmol L^−1^ SO_4_^2−^ from the complete oxidation of 3.75 mmol L^−1^ thiosulfate, only 37% of thiosulfate was oxidized in anaerobic incubations. This indicates the presence of other transformation processes, i.e., the reduction or disproportionation of thiosulfate, commonly found in anoxic sediments [24]. The addition of KNO_3_ (4 mmol L^−1^) to anaerobic microcosms significantly increased the rate of thiosulfate transformation (0.23 ± 0.09 mmol L^−1^ day^−1^; *p* = 0.0474), with a latency of ~4 days (Figure 1A,B). Thereby, complete oxidation to SO_4_^2−^ occurred in close association with a decrease in added NO_3_^−^, while concentrations of both compounds were stable in autoclaved assays until the end of incubation at day 14 (Figure 1C).

### 3.2. Transformation of Total Reduced Inorganic Sulfur (TRIS)

The assessment of total reduced inorganic sulfur (TRIS) in the different incubations revealed significant shifts within the composition of sulfide fractions. While concentrations of free sulfide (H_2_S/HS^−^) were below detection limits (<0.01 mmol L^−1^; Table 1), concentrations of sedimentary sulfides prior to incubation (t0) were dominated by AVS (mainly FeS; 2.62 mmol L^−1^), CRS (mainly FeS_2_; 2.14 mmol L^−1^) and DMFS (mainly S^0^; 1.41 mmol L^−1^) (Figure 1D). In all assays, a significant decrease in AVS (0.009–0.013 mmol L^−1^; *p* < 0.0001) and CRS (*p* = 0.0079) was measured at the end of incubation. The lowest concentrations of CRS were found in aerobic incubations (0.77–0.85 mmol L^−1^) and in anaerobic incubations with the addition of KNO_3_ (0.79 mmol L^−1^). In contrast, there was a tendency for higher CRS concentrations in anaerobic microcosms without KNO_3_ (1.09–1.29 mmol L^−1^), although this difference was not significant. A significant increase (1.68-fold) in the DMFS fraction (mainly S^0^) was measured exclusively in anaerobic incubations with the KNO_3_ addition (2.37 mmol L^−1^; *p* = 0.0159). For all other assays, no significant alterations were observed (Figure 1D).

### 3.3. Comparison to Other Anthropogenic Aquatic Habitats

All studied sediments were anoxic in situ. Cell counts of viable SOB were elevated by two orders of magnitude in tailings material (10^7^ mL^−1^) as compared to samples of the stratified reservoir and the acidic pit lake (10^5^ mL^−1^) (Table 1). Consequently, rates of aerobic and anaerobic thiosulfate oxidation in the oil sands tailings pond were similar to the stratified reservoir Hassel pre-dam and higher than those of the acidic pit lake ML 111. The lowest potential for aerobic thiosulfate oxidation measured in the acidic pit lake (i.e., 0.24 mmol L^−1^ day^−1^) may indicate an effect of thiosulfate instability at low pH. This is supported by lower thiosulfate oxidation rates in ML 111 assays at pH 4 (range 0.05–0.14 mmol L^−1^ day^−1^) compared to pH 6 (range 0.15–0.24 mmol L^−1^ day^−1^), irrespective of the redox condition tested (Table 1). Generally, the different thiosulfate oxidation potentials are all in the same order of magnitude. Calculating cell-specific rates for aerobic thiosulfate conversion, West In-Pit, the Hassel pre-dam and ML 111 yielded 0.02, 5.06 and 1.41 pmol cell^−1^ day^−1^, respectively, and 0.88 pmol cell^−1^ day^−1^ for ML 111 at pH 4.

## 4. Discussion

In this exploratory pilot study, we demonstrated a high indigenous potential for microbially mediated sulfur oxidation in anoxic tailings from an oil sands tailings pond. This is in agreement with considerations that ~81% of produced sulfide is re-oxidized in the pond, which is based on the integration of TRIS accumulation and the prevalent sulfate reduction rates [29]. While the precipitation into iron sulfides is supposed to be the primary mode for initial H_2_S immobilization, we confirmed a strong potential for aerobic microbial sulfur oxidation in the range of rates determined previously [6].

Viable counts of SOB in this study are in the range of those from several acidic sediments [18], Baltic marine sediments [30] and mangrove sediments [31]. The cell-specific rates are an order of magnitude lower than those of [31], which does not imply that the cells were less active, but that our MPN counts were more realistic. However, the cell-specific rates calculated here are again an order of magnitude higher than sulfur transformation rates calculated on the basis of in situ hybridization [32], and thus our viable counts are likely underestimates. The counts in this study were derived from simple acid formation in liquid aerobic media, which does not reflect the diversity and possible site differences in the counted SOB [33]. Although facultative anaerobes might have been active in the moderately oxygenated deep-well plates, the possible presence of base-producing SOB, some of which can also reduce nitrate [33], remains unknown with the present approach. This information would be needed in a comprehensive risk assessment regarding the above-mentioned potentials for ARD generation.

While aerobic microbial sulfide oxidation may occur in oxidized water layers, tailings are highly anoxic environments in situ [4], indicating the potential relevance of anaerobic sulfur oxidation. In our laboratory approach, anaerobic thiosulfate oxidation potentials increased by a factor of 2.5 with the addition of nitrate, compared to assays without NO_3_^−^ or autoclaved controls, which points at microbial sulfur transformation coupled to nitrate reduction. In natural environments, SOB play a prominent role in biogeochemical sulfur cycling and comprise phylogenetically and metabolically diverse organisms that transform a variety of reduced sulfur compounds and may utilize Fe(III) or nitrate as an alternative electron acceptor under anoxic conditions [15,24]. Moreover, sulfur autotrophic denitrification is widely acknowledged as an alternative strategy to improve nitrogen removal performance for wastewater reclamation [16]. Adversely, dissimilatory nitrate reduction may be associated with the production of toxic nitrite, nitric oxide, a recognized mutagen and powerful greenhouse gas, or ammonia [34]. In freshwater sediments, the concentration of free sulfide governed the type of nitrate reduction, with denitrification at low free sulfides, and dissimilatory nitrate reduction to ammonia (DNRA) or incomplete denitrification to gaseous nitrogen oxides at high free sulfides [35]. Local free sulfide concentrations in WIP of 7–18 mg L^−1^ [36] correspond to 218–561 µmol L^−1^ and are thus 4 to 11 times higher than the low values defined by [35]. Formation of gaseous nitrogen oxides or ammonia may therefore occur at least sporadically.

Interestingly, SOB cell counts and rates of nitrate-dependent thiosulfate oxidation in the tailings sample were similar or even higher compared to counts and rates of anoxic sediments of other aquatic habitats in the present study. Likewise, comparison with the existing literature demonstrates that microbial potentials measured in tailings are higher than thiosulfate transformation rates determined in freshwater sediments (0.028 mmol L^−1^ day^−1^) [12] or in the same range (0.06–1.65 mmol L^−1^ day^−1^) [13,37]. This also applies for marine ecosystems (0.026–1.01 mmol L^−1^ day^−1^) [38]. Although nitrate concentrations are typically low in oil sands tailings ponds [1] and other anoxic sediments, MPN estimates and molecular analyses from anoxic tailings revealed an abundance of facultative denitrifying microbes, which may also thrive on fermentative metabolism and hydrocarbon degradation [3,4,7,39]. Similarly, previous estimates showed that ~17% of the viable sulfur-oxidizing community in anoxic tailings is heat resistant or able to form spores [8]. To date, only few sulfur-oxidizing bacilli have been isolated from polluted water [40]; thus, this observation deserves further study. Moreover, the slightly alkaline pH and richness in organic matter in two of the studied habitats suggest that specific heterotrophic SOB [41] might also be involved. Importantly, the addition of nitrate to anaerobic incubations resulted in a significant increase in the DMFS fraction (mostly elemental sulfur) as a potential intermediate during microbial pyrite oxidation [40], accompanied by a decrease in CRS. Elemental sulfur can be further oxidized to sulfate by many lithotrophic SOB and also, to some extent, by heterotrophic SOB [41]. Beside the chemical oxidation, microbial activities may significantly increase the rate of sulfide mineral oxidation in ARD sites, which leads to acid production and heavy metal release [10,42]. Furthermore, pyrite oxidation with nitrate in aquifers is well known as a driver of poor water quality and acidification in groundwaters [43]. In line with our observations, molecular profiling of enrichment cultures at low pH identified various iron- and sulfur-oxidizing microorganisms in oil sands tailings material, which are related to ARD generation in natural habitats [10,44].

Following oxygenation during development of an end pit lake, nitrogen cycling develops [17], and the potential for aerobic sulfur oxidation increases. Even at the suboxic oxygen/ammonia transition zone, nitrification/denitrification by specific microbes is possible [45]. Hence, with respect to the establishment of a future end pit lake, our data indicate that SOB may also accelerate the rate of acid production from sulfide minerals and contribute to the generation of ARD under appropriate conditions.

## 5. Conclusions

Oil sands tailings ponds harbor a high potential for anaerobic microbial thiosulfate oxidation, with rates similar to or higher than those detected in other aquatic environments. Sedimentary sulfide alterations are indicative of microbially catalyzed sulfide mineral oxidation, which implicates an indigenous potential for the generation of acid rock drainage. Moreover, locally high free sulfide concentrations may result in production of gaseous nitrogen oxides or ammonia. Therefore, the microbes involved in combined sulfur and nitrogen cycling in these ponds and their physiological requirements should be studied further.

## Figures and Tables

**Table 1:** Biogeochemical characteristics of anoxic sediments from anthropogenic aquatic habitats.

Habitat	Tailings Pond (West In-Pit)	Stratified Reservoir (Hassel Pre-Dam)	Acidic Pit Lake (ML 111)
**Location**	Fort McMurray, AB, Canada	Hasselfelde, Germany	Lauchhammer, Germany
Depth of tailings/water column at sampling site [m]	12	12	10.2
**Chemistry**			
pH	7.5–8.5	7.2–8.0	2.6
DOC [mg L^−1^]	79	8.4–13.2 [25]	36–78 [26]
NH_4_-N [mg L^−1^]	2.26	2.5–5.6 [*]	2.5–4.3 [27]
NO_3_-N [mg L^−1^]	<0.04	<0.04	2.5–4.3 [27]
SO_4_^2−^ [mg L^−1^]	6.08	19.8	1412
H_2_S [mmol L^−1^]	<0.01	n.a.	0 [28]
Total reactive iron [mg L^−1^]	3158	1786–4576 [25]	1396–12,566 [26]
Ferrous iron [mg L^−1^]	3272	1786–3627 [25]	1396–10,053 [26]
Dissolved iron [mg L^−1^]	0.07	3.9–13.8 [25]	363 [26]
**Microbiology**			
SOB MPN [cells mL^−1^]	5.4 × 10^7^	1.6 × 10^5^	1.7 × 10^5^
Thiosulfate oxidation potentials			
Aerobic [mmol L^−1^ d^−1^]	1.21	0.81	0.24 (pH 6)
			0.15 (pH 4)
Anaerobic [mmol L^−1^ d^−1^]	0.09	n.a.	n.a.
Anaerobic + KNO_3_ [mmol L^−1^ d^−1^]	0.23	0.24	0.15 (pH 6)
			0.05 (pH 4)

n.a. not analyzed. * Kurt Friese; personal communication.

## Data Availability

Raw data are available from the corresponding author upon reasonable request, preferentially by e-mail: katrin.wendt-potthoff@ufz.de.
